# Association between atherogenic index of plasma and prehypertension or hypertension among normoglycemia subjects in a Japan population: a cross-sectional study

**DOI:** 10.1186/s12944-023-01853-9

**Published:** 2023-06-29

**Authors:** Mingjuan Tan, Yongliang Zhang, Ling Jin, Youli Wang, Weiwei Cui, Lubanga Nasifu, Bangshun He

**Affiliations:** 1 Department of Laboratory Medicine, Nanjing First Hospital, Nanjing Medical University, Nanjing, 210006 Jiangsu China; 2 Nanjing First Hospital, Nanjing Medical University, Nanjing, 210006 Jiangsu China; 3 Department of Biology, Muni University, Arua, Uganda

**Keywords:** Atherogenic index of plasma (AIP), Prehypertension, Hypertension, Normoglycemia

## Abstract

**Objective:**

The atherogenic index of plasma (AIP), consisting of triglycerides and high-density lipoprotein cholesterol, is applied to estimate the cardiovascular disease risk. The evidence regarding the association between AIP and prehypertension or hypertension remains inconclusive. This study was conducted to investigate the association of AIP and prehypertension or hypertension in normoglycemic subjects in Japan.

**Methods:**

In the present cross-sectional study, 15,453 normoglycemic participants aged 18 years or older in Gifu, Japan, were evaluated. The selected participants were separated into four groups in the light of AIP quartiles, ranging from the lowest quartile (Q1) to the highest quartile (Q4). And the association between AIP and prehypertension or hypertension was explored with multivariate logistic regression by gradually adjusting model.

**Results:**

Among the 15,453 participants, aged of 43.7 ± 8.9 years, and of whom 45.5% were females, the prevalence rates of prehypertension or hypertension were 27.68% (4,278) and 6.23% (962) respectively. In multivariate logistic regression analyses, participants in the highest AIP quartile had an increase risk in prehypertension and hypertension, compared with participants the lowest one, the odds ratios (OR) were 1.15 (95%CI: 1.00–1.13, *P* = 0.045) for prehypertension and 1.54 (95%CI:1.16–2.04, *P* = 0.003) for hypertension after adjusting confounders. In subgroup analyses, the high risk of hypertension was also observed for female participants in the highest AIP quartile (Q4) (OR = 2.19, 95%CI: 1.37–3.49, *P* = 0.001), especially between the ages of 40 and 60 years (OR = 2.20, 95%CI: 1.24–3.88, *P* = 0.007).

**Conclusions:**

Higher AIP is significantly and positively associated with the risk of prehypertension or hypertension in normoglycemic subjects in Gifu, Japan, which was more pronounced in the female population, especially between the years of 40 and 60.

**Supplementary Information:**

The online version contains supplementary material available at 10.1186/s12944-023-01853-9.

## Introduction

Hypertension is a crucial preventable risk factor for cardio-cerebral vascular diseases and all-cause mortality worldwide [1]. It has been estimated that numerous stroke and ischemic heart disease morbidity and mortality are attributable to hypertension. And 14% of total deaths were caused by systolic blood pressure above 140 mmHg [2]. Furthermore, it is now explicit that the risk of CVD starts from systolic blood pressure well below 140 mmHg. Previous study reported that it existed regional, age and gender differences in prevalence of hypertension [3]. The incidence of hypertension is higher in males than in females before 45 years old, and with similar rates between the ages of 45 and 64. Afterwards, the incidence of hypertension in females is higher than that in males [4]. By 2016, hypertension had become the main risk factor for all-cause mortality in females [5]. Therefore, finding an easily accessible biomarker in clinical practice may contribute to primary prevention and management strategies for hypertension.

Prehypertensive and hypertensive individuals always suffer a long-term concomitant state of dyslipidemia [6], which characteristic is aberrant serum concentration of triglycerides, cholesterol, or both, get involved abnormalities in the associated lipoproteins. Usually, it shows that the concentration of triglycerides (TG), total cholesterol (TC) and low-density lipoprotein cholesterol (LDL-C) aberrantly increase, while high-density lipoprotein cholesterol (HDL-C) decrease [7]. Among these, LDL-C is considered the main therapeutic target [8]. However, after lowering LDL-C to the recommended level, there is still about 50% residual cardiovascular risk [9]. Therefore, it is not sufficient to diagnose risk stratification for cardiovascular disease based on a single lipid marker alone [10].

The atherogenic index of plasma (AIP), calculated with the logarithm of the TG/HDL-C, was observed to be correlated closely with LDL-C particle size [11]. It shows the dynamic levels between serum TG and HDL-C, revealing whether the possible direction of intravascular lipid transfer is the carcinogenic LDL-C or the beneficial HDL-C [12]. Several studies have shown that AIP was associated with atherosclerosis and serious cardiovascular events [13, 14]. Other researches indicated that AIP was also associated with obesity [15], diabetes mellitus [16], and metabolic syndrome [17]. However, to date, there are limited studies on the relation of AIP with hypertension, some of which revealed discordant results. A prospective study in Turkey showed that AIP and hypertension varied by sex, with a significantly weaker association in females than in males [18]. Another longitudinal study also showed that in Taiwan, China, the association of AIP with hypertension was stronger in men than in women [16]. However, a large cross-sectional study observed no significant relevance between AIP and hypertension among adults without type 2 diabetes mellitus (T2DM) in China [19]. So, the evidence regarding the association between AIP and hypertension is still contradictory and uncertain, especially in different sex, age and ethnicity of population. Such, we aim to estimate the association between AIP and prehypertension or hypertension in Japanese normoglycemic individuals in a sex- and age-specific manner using a relevant database.

## Methods

### Data source

The current data came from the DATADRYAD database (https://datadryad.org/stash), courtesy of the Dryad data package (Okamura, Takuro, 2019) [20]. Participant demographic characteristics and baseline indicators included gender and age; lifestyles: smoking, alcohol and exercise; routine examination results: body mass index (BMI), weight, waist circumference (WC), fatty liver, diastolic blood pressure (DBP), systolic blood pressure (SBP); laboratory testing indicators: g-glutamyl transpeptidase (GGT), aspartate transaminase (AST), HDL-C, TC, alanine aminotransferase (ALT), hemoglobin A1c (HbA1c), TG, and fasting plasma glucose (FPG).

### Study participants and study design

The participants in present cross-sectional analysis were from NAGALA (NAfld in the Gifu Area, Longitudinal Analysis) database [20], a health examination item was performed at Murakami Memorial Hospital (Gifu, Japan). This project was established in 1994 to explore chronic diseases and their risk factors. The original authors of this data extracted cases from individuals (aged > 18 years) who take part in the health program from 2004 to 2015 to research the impact of obesity phenotype on T2DM. The exclusion criteria for the raw data were as follows: (1) Missing covariates including height, exercise, alcohol, and abdominal ultrasonography; (2) individuals with alcoholic fatty liver and viral hepatitis for positive of hepatitis C antibody or hepatitis B antigen; (3) Men and women who consumed more than 60 g and 40 g of alcohol per day, respectively; (4) any drug use at baseline; (5) diabetes diagnosis and FPG > 6.1 mmol/L. In current study, participants with missing data of HDL-C were ruled out. Ultimately, this study included 15434 participants. The Ethics Committee of Murakami Memorial Hospital granted ethical approval, and each participant received a written informed consent form.

### Data collection and measurements

The medical history and lifestyles of individuals in this database were collected through a standard questionnaire. The drinking situation including: no or little drinking, light, moderate, and heavy drinking [20, 21]. Smoking status including: never smoked, ever smoked, or currently smoking [20]. In addition, participants were divided into those who did not exercise and those who exercised regularly, i.e., those who regularly performed any type of exercise > 1 × /week [22]. Diagnosis of steatohepatitis based on abdominal ultrasound [23]. The original formula for AIP is Log(TG/HDL-C) [11] and due to the low order of magnitude, we used the natural logarithm transformation Ln(TG/HDL-C) in this study. The selected participants were separated into four groups in the light of AIP quartiles, ranging from the lowest quartile (Q1) to the highest quartile (Q4). Prehypertension or hypertension assessment was performed in conformity to the Japanese Hypertension Guidelines (JSH 2019) [24]. The definition of hypertension is SBP ≥ 140 and/or DBP ≥ 90 mmHg, and prehypertension is SBP 130–139 and/or DBP 80–89 mmHg and SBP120-129 mmHg and DBP < 80 mmHg.

### Statistical analysis

The data were divided into continuous and categorical variables, expressed as mean and standard deviation (SD) for normal distribution, median and interquartile range (IQR) for skewness, and frequency or percentage for categorical variables. The Mann–Whitney test was used for continuous variables and the Chi-squared test was used for categorical variables for comparison between groups in this analysis. Multivariate logistic regression analyses were performed to assess the degree of association of AIP and prehypertension or hypertension, presented as odds ratios (OR) and corresponding 95% confidence intervals (CI). Both unadjusted and multivariate-adjusted models were used, which were as follows; model 1, adjusted for age and sex; model 2, adjusted for model 1 + exercise, smoking and alcohol; model 3, adjusted for model 2 + BMI, HbA1c, TC, fatty liver. In each model analysis, a linear trend test was performed using the median AIP in each quadrant. In this study, the stability of the model was verified by gradually adjusting the confounding factors, which were adjusted according to the following principles (1) The selection of confounding factors was based on previous research findings and clinical restrictions [4, 24]; (2) The variables related with both AIP and hypertension (*P* < 0.05) were chosen, and the odds ratio of matching changed by at least 10% if it was added to the model [25, 26]; (3) All confounding factors related with both AIP and hypertension (*P* < 0.05) were adjusted in sensitivity analysis to determine the stability of the established model including age, sex, smoking, alcohol, exercise, BMI, HbA1c, TC, fatty liver, ALT, AST, GGT, FPG, WC, weight.

In addition, curve fitting was applied to estimate the linear relationship between AIP and prehypertension or hypertension. A subgroup linear regression model was used to identify modification and interaction, and likelihood ratio tests were performed in different subgroups including sex, age, BMI, fatty liver, exercise, smoking, and alcohol status. Stratified analysis was conducted in age and gender stratification. Additional sensitivity analyses were used to assess the stability of the association between AIP and hypertension when redefining hypertension (SBP ≥ 130 and/or DBP ≥ 80 mmHg) in accordance with the new hypertension guidelines [27]. Curve-fitting, stratification analysis and sensitivity analyses were performed using the adjusted confounders in model 3. The analysis for this study was performed with R3.3.2. (http://www.R-project.org, The R Foundation) and Free Statistics software versions 1.7. A bilateral *P* < 0.05 difference was considered statistically significant.

## Results

### Population

**Fig. 1:**
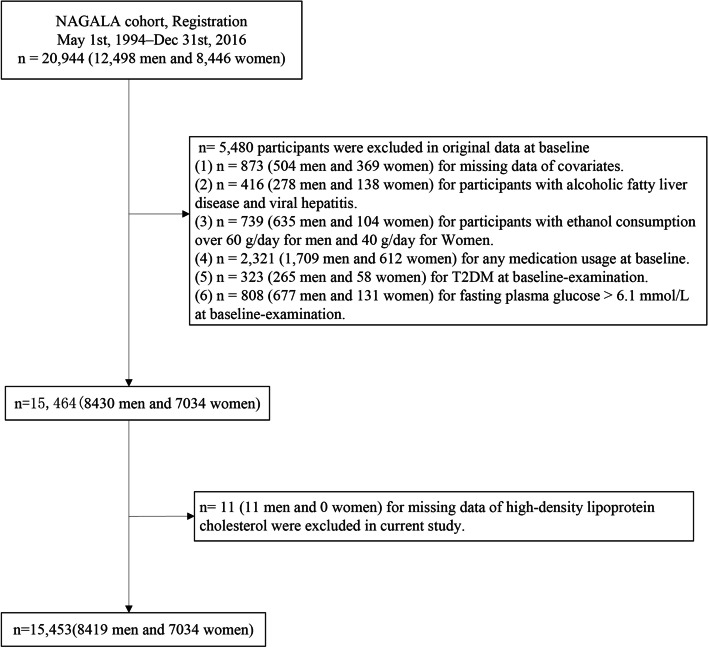
Flow chart of participants selection in this study

The original database included 20,944 participants, of whom 12,498 were male and 8,446 were female. In all 5,480 individuals were excluded for the following reasons: individuals with missing covariates including alcohol, exercise, abdominal ultrasonography and height (*n* = 873); individuals with viral hepatitis and alcoholic fatty liver (*n* = 416); individuals with consuming more than 60g/day of ethanol for men and 40g/day for women (*n* = 739); individuals with any medication use at baseline survey (*n* = 2,321); individuals with T2DM (*n* = 323) or FPG > 6.1 mmol/L (*n* = 808). Individuals with missing data for high-density lipoprotein cholesterol (*n* = 11) were further excluded in this study. Ultimately, this study included 15434 participants, as detailed in participant selection flow chart (Fig. 1).

### Baseline characteristics

Baseline indicators for this study based on AIP quartile groupings are summarized in Table 1. Overall, 15,453 participants aged of 43.7 ± 8.9 years, 7,034(45.5%) participants were females and 8,419 (54.5%) were males, with 14.8% of the female and 85.2% of the males at the highest AIP quartile. The distribution of all the investigated variables, including age, WC, weight, BMI, HDL-C, TC, TG, ALT, AST, FPG, GGT, HbA1c, DBP and SBP show significant differences among groups (Q4 compared with Q1, *P* < 0.001). The proportion of drinking, smoking and having fatty liver gradually increased from Q1 to Q4, along with progressively higher levels of WC, weight, BMI, TC, TG, FPGDBP,SBP, AST, ALT, GGT and HbA1c, but lower levels of HDL-C.

**Table 1:** Baseline characteristics of participants

		**Quartiles of AIP**				
**Variable**	**Total**	**Q1(≤—0.37)**	**Q2(-0.37–0.15)**	**Q3(0.15–0.73)**	**Q4(≥ 0.73)**	***P* value**
Included participants	15,453	3,863	3,863	3,861	3866	
Sex, n (%)						< 0.001
Female	7034 (45.5)	2951 (76.4)	2155 (55.8)	1355 (35.1)	573 (14.8)	
Male	8419 (54.5)	912 (23.6)	1708 (44.2)	2506 (64.9)	3293 (85.2)	
Age, years	43.7 ± 8.9	41.3 ± 8.4	43.4 ± 8.9	45.0 ± 9.1	45.2 ± 8.7	< 0.001
WC, cm	76.5 ± 9.1	70.6 ± 7.0	73.9 ± 7.9	78.0 ± 8.2	83.4 ± 8.0	< 0.001
Weight, kg	60.6 ± 11.6	53.3 ± 8.4	57.7 ± 9.9	62.3 ± 10.5	69.3 ± 11.1	< 0.001
BMI, kg/m2	22.1 ± 3.1	20.3 ± 2.3	21.4 ± 2.6	22.5 ± 2.9	24.3 ± 3.1	< 0.001
Smoking, n (%)						< 0.001
Never	9027 (58.4)	3032 (78.5)	2530 (65.5)	1981 (51.3)	1484 (38.4)	
Past	2949 (19.1)	480 (12.4)	672 (17.4)	840 (21.8)	957 (24.8)	
Current	3477 (22.5)	351 (9.1)	661 (17.1)	1040 (26.9)	1425 (36.9)	
Alcohol, n (%)						< 0.001
None	11802 (76.4)	3264 (84.5)	3007 (77.8)	2854 (73.9)	2677 (69.2)	
Light	1754 (11.4)	317 (8.2)	432 (11.2)	492 (12.7)	513 (13.3)	
Moderate	1357 (8.8)	225 (5.8)	311 (8.1)	361 (9.3)	460 (11.9)	
Heavy	540 (3.5)	57 (1.5)	113 (2.9)	154 (4)	216 (5.6)	
Exercise, n (%)						< 0.001
No	12747 (82.5)	3133 (81.1)	3178 (82.3)	3161 (81.9)	3275 (84.7)	
Yes	2706 (17.5)	730 (18.9)	685 (17.7)	700 (18.1)	591 (15.3)	
Fatty liver, n (%)						< 0.001
No	12716 (82.3)	3781 (97.9)	3577 (92.6)	3164 (81.9)	2194 (56.8)	
Yes	2737 (17.7)	82 (2.1)	286 (7.4)	697 (18.1)	1672 (43.2)	
ALT, IU/L	20.0 ± 14.3	15.1 ± 7.6	17.0 ± 8.9	20.2 ± 11.4	27.5 ± 21.6	< 0.001
AST, IU/L	18.4 ± 8.6	17.1 ± 6.7	17.4 ± 6.0	18.3 ± 7.0	20.9 ± 12.6	< 0.001
GGT, IU/L	20.3 ± 18.1	14.3 ± 9.9	16.8 ± 12.3	21.5 ± 19.0	28.7 ± 24.2	< 0.001
HDL-C, mg/dL	56.5 ± 15.6	71.2 ± 14.7	60.7 ± 11.3	52.2 ± 9.9	42.0 ± 8.2	< 0.001
TC, mg/dL	198.2 ± 33.4	188.2 ± 31.1	193.9 ± 31.4	200.0 ± 33.2	210.7 ± 33.7	< 0.001
TG, mg/dL	65.0(44.0,99.0)	34.0(27.0,41.0)	54.0(47.0,62.0)	79.0(68.0,91.0)	135.0(110.0–173.0)	< 0.001
HbA1, %	5.2 ± 0.3	5.1 ± 0.3	5.1 ± 0.3	5.2 ± 0.3	5.2 ± 0.3	< 0.001
FPG, mg/dL	93.0 ± 7.4	89.6 ± 7.1	91.9 ± 7.2	94.0 ± 7.0	96.3 ± 6.7	< 0.001
SBP, mmHg	114.5 ± 15.0	108.4 ± 13.1	112.0 ± 14.2	116.3 ± 14.5	121.1 ± 14.9	< 0.001
DBP, mmHg	71.6 ± 10.5	67.1 ± 9.3	69.7 ± 9.9	73.0 ± 10.1	76.6 ± 10.3	< 0.001
Prehypertension, n (%)						< 0.001
No	11175 (72.3)	3228 (83.6)	2990 (77.4)	2640 (68.4)	2317 (59.9)	
Yes	4278 (27.7)	635 (16.4)	873 (22.6)	1221 (31.6)	1549 (40.1)	
Hypertension, n (%)						< 0.001
No	14491 (93.8)	3784 (98.0)	3710 (96.0)	3584 (92.8)	3413 (88.3)	
Yes	962 (6.2)	79 (2.0)	153 (4.0)	277 (7.2)	453 (11.7)	
AIP	0.2 ± 0.8	-0.8 ± 0.3	-0.1 ± 0.1	0.4 ± 0.2	1.2 ± 0.4	< 0.001

Data presented are mean ± SD, median (IQR), or N (%); Q1, Q2, Q3, and Q4 are quartiles of the Atherogenic Index of Plasma (AIP), *WC* Waist circumference, *BMI* Body mass index, *ALT* Alanine aminotransferase, *AST* Aspartate aminotransferase, *GGT* Gamma glutamyl transferase, *HDL-C* High-density lipoprotein cholesterol, *TC* Total cholesterol, *TG* Triglyceride, *HbA1*, Hemoglobin A1c, *FPG* Fasting plasma glucose, *SBP* Systolic blood pressure, *DBP* Diastolic blood pressure, *AIP* Atherogenic index of plasma

### Multivariate logistic regression analysis

**Fig. 2:**
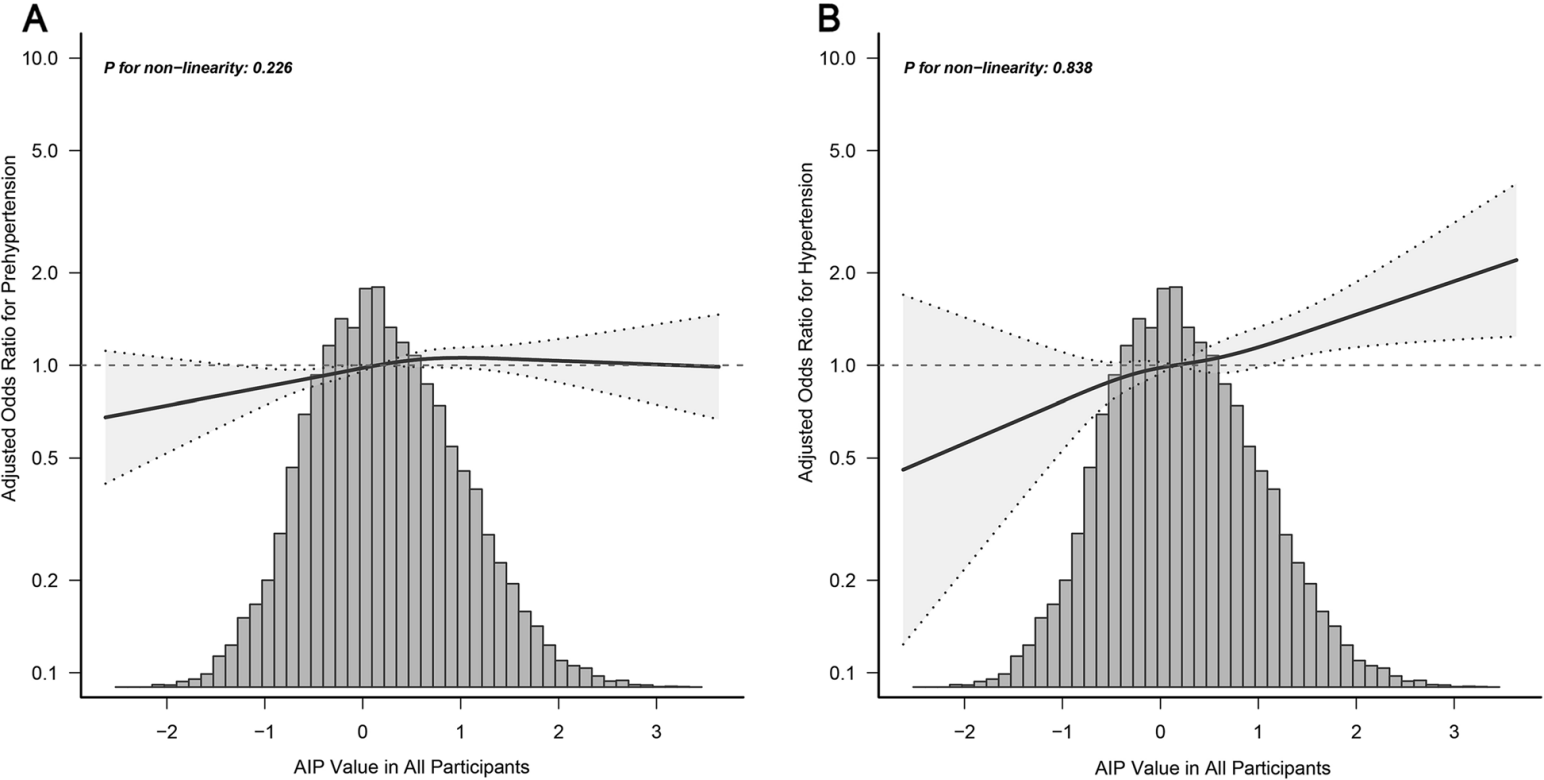
Associations between AIP with prehypertension (**A**) or hypertension (**B**) Odds ratios (OR) were adjusted for age, sex, smoking, alcohol, exercise, body mass index (BMI), hemoglobin A1c(HbA1c), fatty liver, total cholesterol (TC). Both *P* non-linearity > 0.05

AIP was linearly positive associated with prehypertension or hypertension after multivariable adjusted restricted cubic splines as shown in Fig. 2**,** indicating that the risk of prehypertension (Fig. 2A) and hypertension (Fig. 2B) increases with the increase of AIP value in all participants. In addition, curve-fitting was also performed by gender to assess the relationship between AIP and prehypertension or hypertension (Fig. S1). AIP is linearly correlated with prehypertension and hypertension in both male and female, while this correlation is noticeable in female participants (Fig. S1C). The OR and corresponding 95%CI for prehypertension or hypertension according to the AIP quartile are summarized in Table 2. The logistic regression analyses with an unadjusted model revealed a 3.40 time increase in risk of prehypertension (OR = 3.40, 95%CI: 3.05–3.78, *P* < 0.001) and 6.36 time increase in risk of hypertension (OR = 6.36, 95% CI: 4.98–8.11, *P* < 0.001) in Q4 compared to Q1. Similarly, significant increased risks were also observed in the result adjusted with Model1 (adjust for age and sex), Model2 (adjust for Model 1 + smoking, alcohol, exercise), and Model 3(adjust for Model 2 + BMI, HbA1c, fatty liver, TC) (Table 2). When AIP was represented as a continuous variable, a one-unit increase in AIP was associated with an 8% (OR = 1.08, 95% CI:1.01–1.14, *P* = 0.017) and 22% (OR = 1.22, 95%CI:1.09–1.36, *P* < 0.001) increase in the risk of prehypertension and hypertension respectively in adjusted model 3.

**Table 2:** Multivariable logistic regression analyses of the association between AIP and prehypertension and hypertension

**Variable**	**Unadjusted**		**Model 1**		**Model 2**		**Model 3**	
	**OR (95%CI)**	***P* value**	**OR (95%CI)**	***P* value**	**OR (95%CI)**	***P* value**	**OR (95%CI)**	***P* value**
Prehypertension								
AIP	1.80 (1.72–1.89)	< 0.001	1.44 (1.37–1.52)	< 0.001	1.49 (1.41–1.57)	< 0.001	1.08 (1.01–1.14)	0.017
Q1(≤—0.37)	Ref		Ref		Ref		Ref	
Q2(-0.37–0.15)	1.48 (1.32–1.66)	< 0.001	1.23 (1.09–1.38)	0.001	1.24 (1.10–1.40)	< 0.001	1.04 (0.92–1.17)	0.508
Q3(0.15–0.73)	2.35 (2.11–2.62)	< 0.001	1.64 (1.46–1.85)	< 0.001	1.71 (1.52–1.92)	< 0.001	1.20 (1.06–1.35)	0.004
Q4(≥ 0.73)	3.40 (3.05–3.78)	< 0.001	2.09 (1.86–2.35)	< 0.001	2.21 (1.96–2.49)	< 0.001	1.15 (1.00–1.31)	0.045
*P* for trend	1.51 (1.46–1.56)	< 0.001	1.29 (1.24–1.33)	< 0.001	1.31 (1.26–1.36)	< 0.001	1.06 (1.01–1.10)	0.013
Hypertension								
AIP	2.22 (2.04–2.41)	< 0.001	1.83 (1.67–2.00)	< 0.001	1.92 (1.74–2.10)	< 0.001	1.22 (1.09–1.36)	< 0.001
Q1(≤—0.37)	Ref		Ref		Ref		Ref	
Q2(-0.37–0.15)	1.98 (1.50–2.60)	< 0.001	1.57 (1.19–2.08)	0.001	1.60 (1.21–2.11)	0.001	1.19 (0.90–1.59)	0.228
Q3(0.15–0.73)	3.70 (2.87–4.77)	< 0.001	2.44 (1.87–3.17)	< 0.001	2.59 (1.99–3.37)	< 0.001	1.49 (1.13–1.95)	0.004
Q4(≥ 0.73)	6.36 (4.98–8.11)	< 0.001	3.79 (2.92–4.92)	< 0.001	4.12 (3.17–5.35)	< 0.001	1.54 (1.16–2.04)	0.003
* P* for trend	1.83 (1.71–1.95)	< 0.001	1.56 (1.45–1.67)	< 0.001	1.60 (1.49–1.73)	< 0.001	1.15 (1.05–1.24)	< 0.001

Model 1 adjust for age and sex; Model 2 adjust for Model 1 + smoking, alcohol, exercise; Model 3 adjust for Model 2 + BMI, HbA1c, fatty liver, TC, *AIP* As a continuous variable and quartiles variable (Q1, Q2, Q3, and Q4), *AIP* Atherogenic index of plasma, *BMI* Body mass index, *HbA1c* Hemoglobin A1c, *TC* Total cholesterol, *OR* Odds ratio, *CI* Confidence interval

### Subgroup analyses

**Fig. 3:**
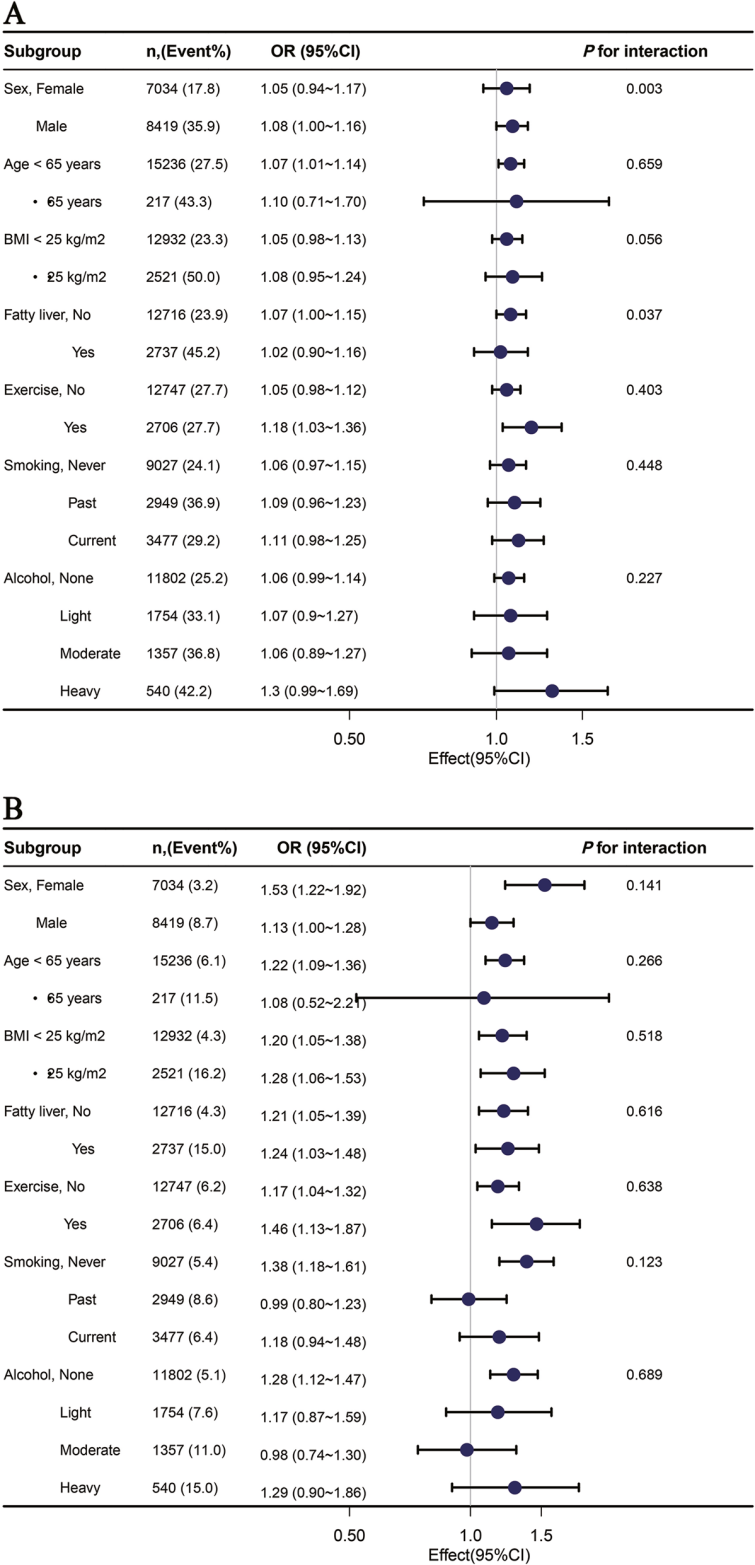
Subgroup analyses of the associations between AIP and prehypertension (**A**) and hypertension (**B**) AIP is continuous variable; Odds ratios (OR) were adjusted for age, sex, smoking, alcohol, exercise, body mass index (BMI), hemoglobin A1c(HbA1c), fatty liver, total cholesterol (TC)

Subgroup analysis was used to explore the correlation of AIP on prehypertension or hypertension in different subgroups (Fig. 3), where prehypertension (Fig. 3A) was the following subgroups: female and male, *P* = 0.003; age < 65 years and ≥ 65 years, *P* = 0.659; BMI < 25 kg/m2 and ≥ 25 kg/m2, *P* = 0.056; having fatty liver or not, *P* = 0.037; regular exercise or not, *P* = 0.403; never smoked and current smoking, *P* = 0.448; none drinking and heavy drinking, *P* = 0.227. Similarly, in subgroup analyses for hypertension (Fig. 3B): female and male, *P* = 0.141; age < 65 years and ≥ 65 years, *P* = 0.266; BMI < 25 kg/m2 and ≥ 25 kg/m2, *P* = 0.518; having fatty liver or not, *P* = 0.616; regular exercise or not, *P* = 0.638; never smoked and current smoking, *P* = 0.123; none drinking and heavy drinking, *P* = 0.689.

### Stratification analysis

Stratification analysis were applied to assess the correlation between AIP and prehypertension or hypertension in different gender and ages (Table S1 and Table S2). In the female population, there were 2.19 times increase in risk of hypertension (OR = 2.19, 95% CI:1.37–3.49, *P* = 0.001) in Q4 compared to Q1 by adjusting model 3. However, such correlation was not found in the male group from Q1 to Q4 (*P* > 0.05) (Table S1). In subgroup aged less than 65 years, AIP in Q4 was significantly associated with prehypertension (OR = 1.45, 95%CI:1.27–1.65, *P* < 0.001) and hypertension (OR = 1.82, 95%CI: 1.38–2.40, *P* < 0.001), but no such correlation was found in people aged over 65 years (*P* > 0.05) (Table S1)**.** Furthermore, between the ages of 40 and 60 for females, the association between AIP in Q4 and hypertension was stronger (OR = 2.20, 95% CI: 1.24–3.88, *P* = 0.007) (Table S2) independent of confounding factors.

### Sensitivity analysis

On the basis of previous finding, clinical constraints and rigorous statistical strategies, gradual adjustments models were used to reduce residual confounding factors in present study [4, 24]. The variables, which were related with both AIP and hypertension (*P* < 0.05), but the matched odds ratio was almost unchanged if it was added to the model [25, 26], was not included in analysis of main results. Therefore, additional sensitivity analysis was performed to verify the stability of the primary outcome by adjusting all confounding factors including model 3 and ALT, AST, GGT, FPG, WC, weight. The association between AIP and hypertension was very stable by adjusted all covariates (Table S3). Furthermore, the result in another sensitive analysis was consistent with the main results when hypertension was redefined according to the new hypertension guidelines [27].

## Discussion

This large retrospective cross-sectional analysis shows that the AIP is positively associated with risk of prehypertension and hypertension in Japanese normoglycemic subjects. Particularly, higher AIP has an obvious correlation with hypertension in the female population, especially between the years of 40 and 60, independent of important covariates and confounders including age, sex, smoking, alcohol, exercise, BMI, fatty liver, HbA1c and TC levels.

For all we know, this the first study reported a stronger association between AIP and the risk of hypertension in female than that in male, which was further conformed by additional sensitivity analysis in current study. One of sensitivity analysis was conducted by adjusting all confounding factors including the covariates in model 3 and ALT, AST, GGT, FPG, WC, weight (Table S3), the other was performed by redefining hypertension according to the new hypertension guidelines (Table S4). However, studies of other populations showed a greater association between AIP and male hypertension risk [16, 18]. Differences in results between current and previous studies may be real, due to the interaction between regional and ethnic differences, lifestyle habits, and other factors [28].

Actually, risk factors for hypertension have been well identified, including obesity, physical inactivity, diabetes, and alcohol use, education, economic and genetic background [4]. Epidemiological studies showed that people who do not exercise had a twofold increased risk of cardiovascular disease, while physically active women have the risk reduced by 50% as compared to sedentary women [28, 29]. Regular light to medium aerobic exercise in females is related with a 5 to 8 mm Hg reduction in blood pressure [30]. However, few people performed regular exercise in this study. In addition, several studies have suggested that multiple sex-specific processes are also mediating the development of hypertension in women [31]. Nevertheless, no significant association was found between AIP and hypertension in the male population in present study. The reasons could be excessive salt intake, obesity and metabolic syndrome was responsible for high prevalence of hypertension in Japanese men [24]. Other studies have reported that high serum levels of TC, LDL-C, and non-HDL-C are related to a rising risk of hypertension in working-age men of Japanese [7].

Often, Hypertension and dyslipidemia co-exist in clinical practice [32]. And, the progressive increase in blood pressure and prevalence of hypertension were related with increased serum lipid concentrations [33, 34]. Possible reasons are that hypertension and dyslipidemia share common pathophysiological mechanisms, such as obesity and release of adipocytokines from the abnormal adipose tissue [35]. The structure and function of arterial vessel walls are affected by dyslipidemia, which promote atherosclerosis and make blood pressure dysregulation [36]. However, the mechanism of dyslipidemia and hypertension in females may be different from that in males due to the physiological cycles and hormonal changes during their life [37]. Estrogen deficiency caused by menopause may lead to metabolic disorders [38], which is a possible explanation for the stronger relationship between AIP and hypertension in females aged between 40 and 60 years in this study. Moreover, our result was partly supported by previous studies that median age for natural menopause is between 48 and 53 years old, with women having significantly higher systolic and diastolic blood pressure [39, 40]. Meanwhile, Dyslipidemia such as reduced HDL-C and elevated LDL-C and TG levels were associated with the menopausal transition [41], and the quality or functional capacity of HDL-C may be undergo alterations in the process [42].

In this study, we also observed that the association of AIP with prehypertension or hypertension was affected with age. Subgroup analyses found that higher AIP only demonstrated significant association with prehypertension or hypertension for participants aged less than 65 years, but not in aged over 65 years. This finding was consistent with a recent study in Taiwan citizens of China [16]. However, there are also several reports with different conclusions about the failure of AIP to maintain the same relationship with other age subgroups. A clinical controlled trial found that AIP was positively associated with cardiovascular disease risk and severity in older male individuals (age ≥ 65 years) [43]. Another study showed that AIP might be a powerful marker to predict the risk of coronary artery disease in Chinese postmenopausal women [13]. But the relationship between AIP and prehypertension or hypertension was not obvious at over 65 in this study, except for the possible influence factors of region, diet, ethnic differences, or due to fewer people older than 65 years in this population. Therefore, it is necessary to further analyze the relationship between AIP and hypertension by expanding the sample size of the population aged over 65 years among different populations in the future.

Notably, no association was found between AIP and prehypertension in females age-stratified analyses in this study. Systolic blood pressure over 140 mmHg was the main cause of death due to hypertension. Yet researches showed that cardiovascular risk begins at least 110 to 115 mmHg, considered to be the theoretical lowest risk level for blood pressure [2]. In Japan, people with blood pressure of (120–139)/ (80–89) mmHg have a higher incidence of cardiovascular disease than those with blood pressure of 120/80 mmHg [44]. So, high normotension was also defined as prehypertension in the present study [24]. It is possible that the definition of prehypertension is too broad, resulting in negative results.

### Strengths and limitations

The following strengths are presented in current study. First, current study had an adequate sample size and credible data sources, making the finding relatively reliable. Second, clinical constraints and rigorous statistical strategies were used to reduce residual confounding factors by gradual adjustments model. Third, Sensitivity analysis and subgroup analysis were conducted in a sex- and age-specific manner to verify the stability of the model.

However, some limitations of this study must be thoughtful. First, based on the nature of observational studies themselves, the results of this study have inherent limitations in eliminating causality. Second, the possible effects of some underlying diseases could not be ruled out in this population, so further population-based prospective studies are required to uncover the mechanisms underline the association between AIP and hypertension. Third, since the study data were obtained from a healthcare program in Gifu, Japan, the applicability of the findings to other races is unclear. Finally, the majority of the population in this study is middle-aged people, with a limited number of elderly people, which may be the reason for the unclear relationship between AIP and hypertension in the elderly population.

## Conclusions

Our study showed a positively linear association between higher AIP and risk of prehypertension or hypertension. The association was stronger in the female population, especially between the years of 40 and 60. Future, AIP may be used as a marker to monitor the risk of hypertension, but more research is needed to verify this.

## Supplementary Information

**Additional file 1:** **Table S1.** Stratification analysis of the association between AIP andprehypertension and hypertension.

**Additional file 2:** **Table S2.** Stratificationanalysis of the association between AIP and hypertension in female groups.

**Additional file 3:** **Table S3.** Sensitivity analysis of the association between AIP andhypertension in different participants groups by adjusted all covariates.

**Additional file 4:** **Table S4.** Sensitivity analysis of the association between AIP andhypertension(130/80mmHg) in different group.

**Additional file 5**: **Fig. S1.** Associations betweenAIP with prehypertension or hypertension by gender.

## Data Availability

The data in current study was obtained from the DATADRYAD database (https://datadryad.org/stash).

## Abbreviations

AIP: The atherogenic index of plasma
CVD: Cardiovascular disease
LDL-C: Low-density lipoprotein cholesterol
TC: Total cholesterol
TG: Triglyceride
HDL-C: High-density lipoprotein cholesterol
WC: Waist circumference
BMI: Body mass index
ALT: Alanine aminotransferase
AST: Aspartate transaminase
GGT: G-glutamyl transpeptidase
HbA1c: Hemoglobin A1c
FPG: Fasting plasma glucose
SBP: Systolic blood pressure
DBP: Diastolic blood pressure
DM: Diabetes mellitus
SD: Standard deviation
IQR: Interquartile range
OR: Odds ratio
CI: Confidence interval
