# Supplementary material for: Association between atherogenic index of plasma and prehypertension or hypertension among normoglycemia subjects in a Japan population: a cross-sectional study
Source: Lipids Health Dis. 2023 Jun 29;22:87. doi: 10.1186/s12944-023-01853-9 (PMC10308786; doi:10.1186/s12944-023-01853-9)
Supplement: Supplementary file 1 — Additional file 1: Table S1. Stratification analysis of the association between AIP andprehypertension and hypertension. [file 12944_2023_1853_MOESM1_ESM.docx]

| **Table S1** Stratification analysis of the association between AIP and prehypertension and hypertension | | | | | |
| --- | --- | --- | --- | --- | --- |
|  |  | **Prehypertension** | | **Hypertension** | |
| **Subgroup** | **Variable, n** | **Adjusted, OR (95%CI)** | ***P* value** | **Adjusted, OR (95%CI)** | ***P* value** |
| Female | AIP,7034 | 1.05 (0.94-1.17) | 0.427 | 1.53 (1.22-1.92) | <0.001 |
|  | Q1, 1758 | Ref |  | Ref |  |
|  | Q2, 1759 | 0.98 (0.75-1.05) | 0.176 | 0.98 (0.64-1.50) | 0.918 |
|  | Q3, 1758 | 1.12 (0.93-1.34) | 0.221 | 2.00 (1.34-2.98) | 0.001 |
|  | Q4, 1759 | 1.02 (0.80-1.30) | 0.863 | 2.19 (1.37-3.49) | 0.001 |
| Male | AIP,8419 | 1.07 (1.00-1.15) | 0.059 | 1.13 (1.00-1.29) | 0.05 |
|  | Q1, 2105 | Ref |  | Ref |  |
|  | Q2, 2104 | 1.05 (0.88-1.27) | 0.577 | 1.30 (0.87-1.94) | 0.207 |
|  | Q3, 2105 | 1.14 (0.95-1.35) | 0.156 | 1.28 (0.87-1.87) | 0.216 |
|  | Q4, 2105 | 1.16 (0.97-1.39) | 0.105 | 1.32 (0.90-1.94) | 0.155 |
| Ages<65 | AIP,15236 | 1.07 (1.01-1.14) | 0.025 | 1.22 (1.09-1.36) | <0.001 |
|  | Q1, 3827 | Ref |  | Ref |  |
|  | Q2, 3817 | 1.14 (1.01-1.28) | 0.035 | 1.23 (0.92-1.64) | 0.155 |
|  | Q3, 3786 | 1.41 (1.25-1.59) | <0.001 | 1.62 (1.24-2.13) | 0.001 |
|  | Q4, 3806 | 1.45 (1.27-1.65) | <0.001 | 1.82 (1.38-2.40) | <0.001 |
| Ages≥65 | AIP,217 | 1.10 (0.71-1.70) | 0.683 | 1.08 (0.53-2.21) | 0.837 |
|  | Q1, 36 | Ref |  | Ref |  |
|  | Q2, 46 | 1.10 (0.43-2.82) | 0.836 | 11.89 (1.10-128.81) | 0.042 |
|  | Q3, 75 | 0.95 (0.40-2.24) | 0.902 | 10.68 (1.07-107.03) | 0.044 |
|  | Q4,60 | 1.14 (0.44-2.91) | 0.791 | 7.62 (0.67-86.04) | 0.101 |
| Adjusted Covariates: age, sex, smoking, alcohol, exercise, BMI, HbA1c, Fatty liver, TC; AIP as a continuous variable and quartiles variable (Q1, Q2, Q3, and Q4); AIP Atherogenic Index of Plasma, BMI body mass index, HbA1c hemoglobin A1c, TC total cholesterol. | | | | | |
