# Supplementary material for: Association between atherogenic index of plasma and prehypertension or hypertension among normoglycemia subjects in a Japan population: a cross-sectional study
Source: Lipids Health Dis. 2023 Jun 29;22:87. doi: 10.1186/s12944-023-01853-9 (PMC10308786; doi:10.1186/s12944-023-01853-9)
Supplement: Supplementary file 2 — Additional file 2: Table S2. Stratificationanalysis of the association between AIP and hypertension in female groups. [file 12944_2023_1853_MOESM2_ESM.docx]

| **Table S2** Stratification analysis of the association between AIP and hypertension in female groups | | | | | |
| --- | --- | --- | --- | --- | --- |
|  |  | **Hypertension** | | |  |
| **Subgroup** | **Variable, n (event%)** | **Unadjusted OR (95%CI)** | ***P* value** | **Adjusted OR (95%CI)** | ***P* value** |
| Age <40 | AIP, 2574 (1.1) | 2.23 (1.3-3.81) | 0.004 | 1.09 (0.57-2.09) | 0.799 |
|  | Q1, 885(0.9) | 1(Ref) |  | 1(Ref) |  |
|  | Q2, 702(0.6) | 0.63 (0.19-2.09) | 0.449 | 0.48 (0.14-1.63) | 0.239 |
|  | Q3, 598(0.7) | 0.74 (0.22-2.46) | 0.621 | 0.45 (0.13-1.57) | 0.212 |
|  | Q4, 389(3.1) | 3.49 (1.41-8.61) | 0.007 | 1.13 (0.37-3.40) | 0.831 |
| Age=40-60 | AIP, 4224 (4.3) | 2.62 (2.11-3.26) | <0.001 | 1.68 (1.3-2.18) | <0.001 |
|  | Q1, 850(1.9) | 1(Ref) |  | 1(Ref) |  |
|  | Q2, 1016(2.7) | 1.42 (0.76-2.66) | 0.269 | 1.15 (0.61-2.16) | 0.672 |
|  | Q3, 1095(2.9) | 1.57 (0.86-2.88) | 0.146 | 1.09 (0.59-2.01) | 0.794 |
|  | Q4, 1263(8.3) | 4.73 (2.77-8.06) | <0.001 | 2.20 (1.24-3.88) | 0.007 |
| Age≥60 | AIP,236 (8.5) | 1.34 (0.66-2.72) | 0.415 | 1.07 (0.5-2.31) | 0.863 |
|  | Q1, 23(8.7) | 1(Ref) |  | 1(Ref) |  |
|  | Q2, 41(7.3) | 0.83 (0.13-5.36) | 0.844 | 0.62 (0.08-4.60) | 0.643 |
|  | Q3, 65(3.1) | 0.33 (0.04-2.52) | 0.287 | 0.28 (0.03-2.29) | 0.237 |
|  | Q4, 107(12.1) | 1.45 (0.30-6.92) | 0.64 | 1.01 (0.19-5.28) | 0.987 |
| Adjusted Covariates: age, smoking, alcohol, exercise, BMI, HbA1c, Fatty liver, TC; AIP as a continuous variable and quartiles variable (Q1, Q2, Q3, and Q4); AIP Atherogenic Index of Plasma, BMI body mass index, HbA1c hemoglobin A1c, TC total cholesterol. | | | | | |
