# Supplementary material for: Association between atherogenic index of plasma and prehypertension or hypertension among normoglycemia subjects in a Japan population: a cross-sectional study
Source: Lipids Health Dis. 2023 Jun 29;22:87. doi: 10.1186/s12944-023-01853-9 (PMC10308786; doi:10.1186/s12944-023-01853-9)
Supplement: Supplementary file 3 — Additional file 3: Table S3. Sensitivity analysis of the association between AIP andhypertension in different participants groups by adjusted all covariates. [file 12944_2023_1853_MOESM3_ESM.docx]

| **Table S3** Sensitivity analysis of the association between AIP and hypertension in different participants groups by adjusted all covariates | | | | | |
| --- | --- | --- | --- | --- | --- |
|  |  | **Hypertension** | | |  |
| **Group** | **Variable, n (Event%)** | **Unadjusted OR (95%CI)** | ***P* value** | **Adjusted OR (95%CI)** | ***P* value** |
| All participants | AIP, 15453 (6.2) | 2.22 (2.04-2.41) | <0.001 | 1.17 (1.05-1.31) | 0.004 |
|  | Q1, 3863 (2.0) | 1(Ref) |  | 1(Ref) |  |
|  | Q2, 3863 (4.0) | 1.98 (1.50-2.60) | <0.001 | 1.16 (0.87-1.55) | 0.302 |
|  | Q3, 3861 (7.2) | 3.70 (2.87-4.77) | <0.001 | 1.42 (1.08-1.87) | 0.012 |
|  | Q4, 3866 (11.7) | 6.36 (4.98-8.11) | <0.001 | 1.45 (1.09-1.93) | 0.011 |
| Female participants | AIP, 7034 (3.2) | 2.77 (2.3-3.34) | <0.001 | 1.49 (1.18-1.87) | 0.001 |
|  | Q1, 2951 (1.5) | 1(Ref) |  | 1(Ref) |  |
|  | Q2, 2155 (2.1) | 1.44 (0.95-2.19) | 0.086 | 0.95 (0.62-1.46) | 0.82 |
|  | Q3, 1355 (6.1) | 4.31 (2.97-6.25) | <0.001 | 1.94 (1.30-2.90) | 0.001 |
|  | Q4, 573 (9.6) | 7.01 (4.67-10.54) | <0.001 | 2.08 (1.30-3.34) | 0.002 |
| Male participants | AIP, 8419 (8.7) | 1.74 (1.57-1.93) | <0.001 | 1.09 (0.96-1.24) | 0.195 |
|  | Q1, 912 (3.8) | 1(Ref) |  | 1(Ref) |  |
|  | Q2, 1708 (6.3) | 1.67 (1.13-2.47) | 0.01 | 1.27 (0.84-1.90) | 0.253 |
|  | Q3, 2506 (7.7) | 2.10 (1.46-3.04) | <0.001 | 1.21 (0.82-1.79) | 0.332 |
|  | Q4, 3293 (12.1) | 3.44 (2.42-4.91) | <0.001 | 1.24 (0.84-1.83) | 0.276 |
| Age=40-60 (female) | AIP, 4224 (4.3) | 2.62 (2.11-3.26) | <0.001 | 1.65 (1.27-2.15) | <0.001 |
|  | Q1, 850 (1.9) | 1(Ref) |  | 1(Ref) |  |
|  | Q2, 1016 (2.7) | 1.42 (0.76-2.66) | 0.269 | 1.13 (0.60-2.13) | 0.713 |
|  | Q3, 1095 (2.9) | 1.57 (0.86-2.88) | 0.146 | 1.03 (0.55-1.92) | 0.922 |
|  | Q4, 1263 (8.3) | 4.73 (2.77-8.06) | <0.001 | 2.08 (1.17-3.69) | 0.012 |
| Adjusted covariates: age, sex, smoking, alcohol, exercise, BMI, HbA1c, Fatty liver, TC, ALT, AST, GGT, FPG, WC, weight; AIP as a continuous variable and quartiles variable (Q1, Q2, Q3, and Q4); AIP Atherogenic Index of Plasma, OR odds ratio, BMI body mass index, HbA1 hemoglobin A1c, TC total cholesterol, ALT alanine aminotransferase, AST aspartate aminotransferase, GGT gamma glutamyl transferase, FPG fasting plasma glucose, WC waist circumference. | | | | | |
