# Supplementary material for: Association between atherogenic index of plasma and prehypertension or hypertension among normoglycemia subjects in a Japan population: a cross-sectional study
Source: Lipids Health Dis. 2023 Jun 29;22:87. doi: 10.1186/s12944-023-01853-9 (PMC10308786; doi:10.1186/s12944-023-01853-9)
Supplement: Supplementary file 4 — Additional file 4: Table S4. Sensitivity analysis of the association between AIP andhypertension(130/80mmHg) in different group. [file 12944_2023_1853_MOESM4_ESM.docx]

| **Table S4** Sensitivity analysis of the association between AIP and hypertension(>130/80mmHg) in different groups | | | | | |
| --- | --- | --- | --- | --- | --- |
|  |  | **Hypertension (**130/80 mmHg**)** | | |  |
| **Group** | **Variable, n (Event%)** | **Unadjusted OR (95%CI)** | ***P* value** | **Adjusted OR (95%CI)** | ***P* value** |
| All participants | AIP, 15453 (22.9) | 2.21 (2.10-2.33) | <0.001 | 1.20 (1.12-1.28) | <0.001 |
|  | Q1, 3863 (10.6) | 1(Ref) |  | 1(Ref) |  |
|  | Q2, 3863 (16.4) | 1.66 (1.45-1.90) | <0.001 | 1.04 (0.90-1.20) | 0.574 |
|  | Q3, 3861 (26.7) | 3.07 (2.71-3.48) | <0.001 | 1.32 (1.14-1.51) | <0.001 |
|  | Q4, 3866 (37.9) | 5.15 (4.56-5.81) | <0.001 | 1.34 (1.15-1.56) | <0.001 |
| Female participants | AIP, 7034 (12.8) | 2.34 (2.10-2.60) | <0.001 | 1.27 (1.12-1.44) | <0.001 |
|  | Q1, 2951 (8.0) | 1(Ref) |  | 1(Ref) |  |
|  | Q2, 2155 (10.1) | 1.30 (1.07-1.58) | 0.008 | 0.88 (0.71-1.08) | 0.209 |
|  | Q3, 1355 (20.8) | 3.04 (2.52-3.66) | <0.001 | 1.42 (1.15-1.75) | 0.001 |
|  | Q4, 573 (28.4) | 4.59 (3.67-5.76) | <0.001 | 1.42 (1.09-1.85) | 0.01 |
| Male participants | AIP, 8419 (31.4) | 1.67 (1.57-1.78) | <0.001 | 1.16 (1.07-1.25) | <0.001 |
|  | Q1, 912 (19.1) | 1(Ref) |  | 1(Ref) |  |
|  | Q2, 1708 (24.4) | 1.37 (1.12-1.67) | 0.002 | 1.14 (0.92-1.40) | 0.234 |
|  | Q3, 2506 (29.8) | 1.80 (1.50-2.17) | <0.001 | 1.24 (1.02-1.52) | 0.032 |
|  | Q4, 3293 (39.5) | 2.77 (2.32-3.31) | <0.001 | 1.30 (1.06-1.6) | 0.011 |
| Age65<65 | AIP, 15236 (22.7) | 2.23 (2.12-2.35) | <0.001 | 1.19 (1.11-1.27) | <0.001 |
|  | Q1, 3827 (10.4) | 1(Ref) |  | 1(Ref) |  |
|  | Q2, 3817 (16.2) | 1.66 (1.45-1.90) | <0.001 | 1.03 (0.89-1.19) | 0.664 |
|  | Q3, 3786 (26.5) | 3.11 (2.74-3.53) | <0.001 | 1.30 (1.13-1.50) | <0.001 |
|  | Q4, 3806 (37.8) | 5.23 (4.63-5.92) | <0.001 | 1.32 (1.14-1.54) | <0.001 |
| Age65≥65 | AIP, 217 (36.9) | 1.23 (0.83-1.83) | 0.293 | 1.25 (0.79-1.98) | 0.348 |
|  | Q1, 36 (30.6) | 1(Ref) |  | 1(Ref) |  |
|  | Q2, 46 (37.0) | 1.33 (0.53-3.37) | 0.545 | 1.49 (0.54-4.12) | 0.447 |
|  | Q3, 75 (34.7) | 1.21 (0.51-2.83) | 0.667 | 1.59 (0.63-4.05) | 0.328 |
|  | Q4, 60 (43.3) | 1.74 (0.73-4.16) | 0.215 | 1.9 (0.68-5.28) | 0.217 |
| Adjust covariates: age, sex, smoking, alcohol, exercise, BMI, HbA1c, Fatty liver, TC；AIP as a continuous variable and quartiles variable (Q1, Q2, Q3, and Q4); AIP Atherogenic Index of Plasma, BMI body mass index, HbA1c hemoglobin A1c, TC total cholesterol; Hypertension is defined by SBP ≥130 and/or DBP ≥80 mmHg according to the new ACC/AHA hypertension guidelines (ACC American College of Cardiology, AHA American Heart Association). | | | | | |
