# Supplementary figures and images for: Association between atherogenic index of plasma and prehypertension or hypertension among normoglycemia subjects in a Japan population: a cross-sectional study
Source: Lipids Health Dis. 2023 Jun 29;22:87. doi: 10.1186/s12944-023-01853-9 (PMC10308786; doi:10.1186/s12944-023-01853-9)

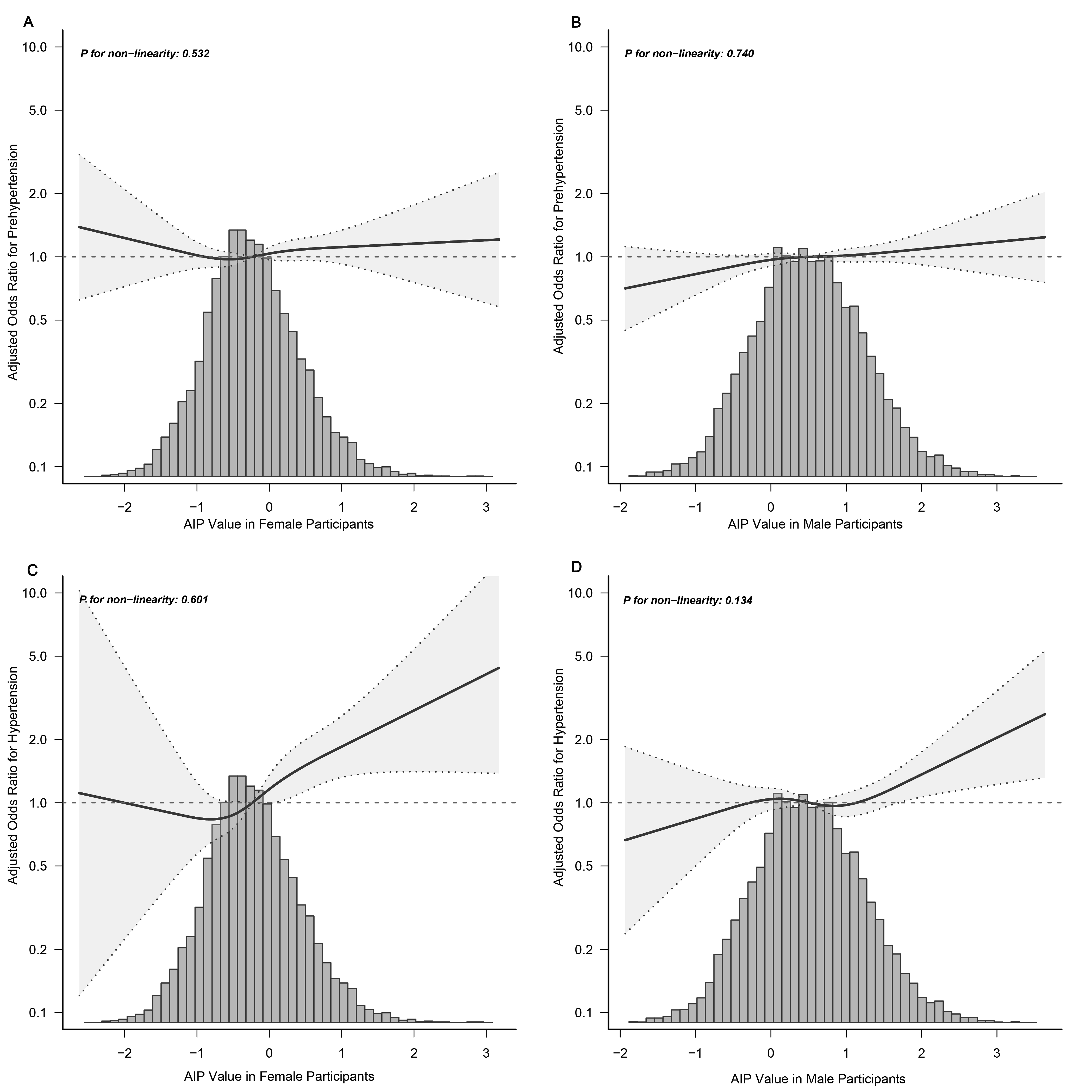

Supplement: Supplementary file 5 — Additional file 5: Fig. S1. Associations betweenAIP with prehypertension or hypertension by gender. [file 12944_2023_1853_MOESM5_ESM.jpg]
